# Trans‐Coumaryl acetate mediates GRK5/NF‐κB/Nrf2 signaling axis to ameliorate septic acute kidney injury

**DOI:** 10.1002/ccs3.70044

**Published:** 2025-09-04

**Authors:** Jie Liu, Yugang Deng, Kunyang Lei, Yaqi Li, Siwei Ma

**Affiliations:** ^1^ Department of Urology Henan Provincial People's Hospital (People's Hospital of Zhengzhou University) Zhengzhou Henan China; ^2^ School of Clinical Medicine Henan University Zhengzhou Henan China

**Keywords:** G protein‐coupled receptor kinase 5, mitochondria damage, NF‐κB/Nrf2 pathway, septic acute kidney injury, Trans‐Coumaryl acetate

## Abstract

Trans‐Coumaryl acetate (T‐CA) is formed by the esterification of coumarin with acetic acid and belongs to the reprogramming products of aromatic amino acid and fatty acid metabolism. Currently, the impact of T‐CA on the progression of septic acute kidney injury (SAKI) and its underlying mechanisms are not clear. A lipopolysaccharide (LPS)‐treated HK‐2 cell injury model was constructed, and a mouse SAKI model was constructed using a cecum ligation and puncture method. The impacts of T‐CA on HK‐2 cell survival and cytotoxicity were examined using a Cell Counting Kit‐8 assay and lactate dehydrogenase kit. Inflammatory factors, Superoxide dismutase (SOD), glutathione (GSH), malondialdehyde (MDA), reactive oxygen species (ROS), adenosine 5′‐triphosphate (ATP), and mitochondrial membrane potential levels were measured using different kits. Apoptosis was identified using Hoechst 33258 and Terminal Deoxynucleotidyl Transferase mediated dUTP Nick‐End Labeling (TUNEL) staining. Changes in renal histopathological injury and indicator protein expression in SAKI mice were observed by transmission electron microscopy and pathological staining. Western blot was used to assess the levels of G protein‐coupled receptor kinase 5 (GRK5)/NF‐κB/nuclear factor erythroid‐2 related factor 2 (Nrf2) pathway, apoptosis and mitochondrial damage‐related proteins. T‐CA (2.5–20 μM) treatment for 24 h did not negatively impact HK‐2 cell viability. In vitro, T‐CA attenuated LPS‐induced HK‐2 cell injury while reducing cell mortality, inflammatory factor levels and oxidative stress injury. In vivo, intraperitoneal injection of 40 mg/kg of T‐CA attenuated renal histopathological damage and apoptosis in SAKI mice. Additionally, T‐CA reduced mitochondrial damage, MDA and ROS levels, and increased SOD, GSH, and ATP levels. T‐CA down‐regulated GRK5 protein, hindered NF‐κB activation and activated Nrf2 pathway, and NF‐κB activator Phorbol 12‐myristate 13‐acetate (PMA), Nrf2 inhibitor ML385 treatment and overexpression of GRK5 weakened the protective effect of T‐CA in SAKI model. T‐CA has the potential to improve SAKI by inhibiting mitochondrial dysfunction, increase cell viability and ameliorate renal injury through the GRK5/NF‐κB/Nrf2 pathway in SAKI models.

## Graphical abstract

A mouse septic acute kidney injury (SAKI) model by cecum ligation and puncture method and an HK‐2 cell injury model through lipopolysaccharide‐induced were used to investigate how trans‐Coumaryl acetate (T‐CA) influences SAKI and its underlying mechanisms. T‐CA down‐regulates GRK5 expression, suppresses NF‐κB activation, and activates the nuclear factor erythroid‐2 related factor 2 signaling pathway, thereby reducing inflammation, oxidative stress, and mitochondrial dysfunction to ameliorate septic acute kidney injury.

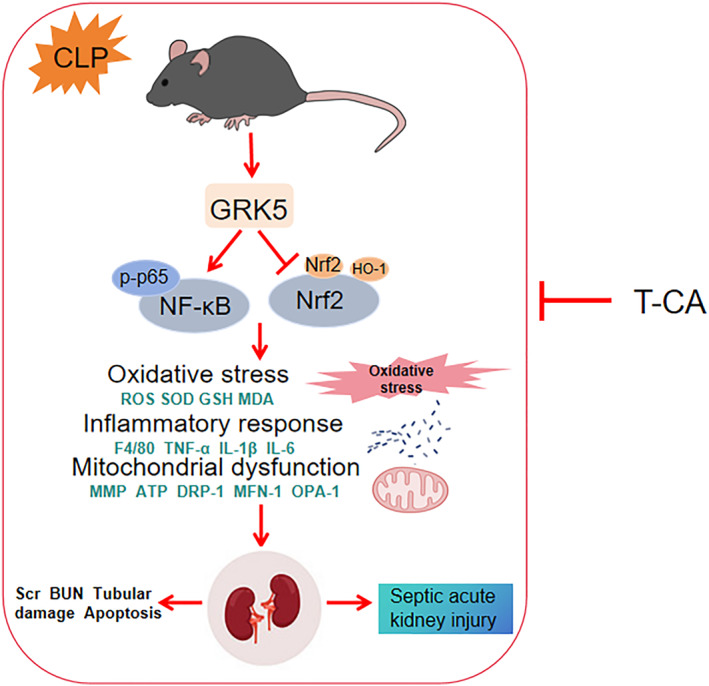

## 1 INTRODUCTION

Sepsis occurs due to infection, in which a large number of inflammatory mediators are released so that the body loses its normal immunoregulatory function, which can cause different degrees of damage and dysfunction in multiple organs and tissues.^1^ According to reports, sepsis impacts over 19 million individuals worldwide annually, of whom 6 million die, with a case‐fatality rate of more than one‐quarter.^2^ Sequential organ failure is the main manifestation of sepsis, in which septic acute kidney injury (SAKI) is a main reason of the poor prognosis of patients with sepsis, accounting for about 50% of all acute kidney injury causes.^3^, ^4^ The primary clinical approach for SAKI is a renal replacement therapy; however, it does not significantly improve local perfusion and survival, especially in the early stages, and increases the number of dialysis sessions and bloodstream infections in patients.^5^, ^6^ For a long time, the number of deaths and poor prognosis due to SAKI have not been effectively controlled, and the underlying pathogenesis of SAKI has not been fully understood. Therefore, there is an urgent need to explore the pathophysiologic mechanisms in SAKI with a view to establish a foundation for its diagnosis and therapeutic approaches.

Previous studies have suggested that immune, inflammatory, and oxidative stress‐mediated injury and acute tubular necrosis are important pathophysiologic mechanisms leading to the development of SAKI, but these theories do not fully explain the pathophysiologic course of SAKI.^7^, ^8^ Recent studies also point to an important role for metabolic reprogramming in SAKI disease progression.^9^, ^10^ Metabolic reprogramming in SAKI may lead to reduced adenosine 5′‐triphosphate (ATP) synthesis capacity and phenotypic transformation of renal tubular epithelial cells, thereby down‐regulating transporter proteins, leading to disruption of tubular ion transport, impairing metabolic activity, and triggering mitochondrial dysfunction.^11^, ^12^ It can be seen that sepsis can induce metabolic reprogramming, causing mitochondrial dysfunction, and a variety of active metabolites are not only the material basis of metabolism in this process, but also participate in the regulation of the progression of SAKI as protein activity regulating substances.^13^ Trans‐Coumaryl acetate (T‐CA) is formed by the esterification of coumarin with acetic acid, which belongs to the reprogramming product of aromatic amino acid and fatty acid metabolism, and its molecular formula is C_11_H_12_O_3_. T‐CA and its analogs have been documented to exhibit a range of biological functions, such as antitumor,^14^ regulating insulin secretion,^15^ and improving glucose homeostasis.^16^ Daphnetin, also a natural coumarin derivative, has been found to reduce the release of pro‐inflammatory cytokines in the serum of septic lung injury mice, thus attenuating lung pathology, suggesting its therapeutic potential for septic lung injury.^17^ However, the effect of T‐CA on SAKI has not yet been reported.

G protein‐coupled receptor kinase 5 (GRK5) is responsible for transducing extracellular signals into the cell and affecting the corresponding biological progression by modulating downstream signaling pathways.^18^ GRK5 is essential in the progression of many diseases, such as those involved in myocardial infarction in heart failure,^19^ renal fibrosis,^20^ and synovial inflammation.^21^ Furthermore, GRK5 knockout mice were significantly more likely to survive and had reduced levels of pro‐inflammatory cytokines in a sepsis model triggered by cecum ligation and puncture (CLP).^22^ This implies that GRK5 could serve as a promising molecular target for SAKI. NF‐κB is a crucial transcription factor involved in a variety of biological processes and is responsible for regulating immune response.^23^, ^24^ Nuclear factor erythroid‐2 related factor 2 (Nrf2) is a key regulator of the cellular antioxidant response and encodes antioxidant, anti‐inflammatory, and detoxification protein genes that improve cellular tolerance to oxidative stress.^25^ Jin et al. demonstrated that NF‐κB/Nrf2 pathway is crucial in regulating oxidative stress and inflammatory damage in renal tissue in the mouse model of arsenic trioxide‐induced renal injury.^26^ Notably, GRK5 activates NF‐κB signaling.^22^, ^27^ Therefore, we constructed a mouse SAKI model using the CLP method and an HK‐2 cell injury model through lipopolysaccharide (LPS)‐induction to investigate how T‐CA influences SAKI and its underlying mechanisms, and delve deeper into its possible function in regulating the GRK5/NF‐κB/Nrf2 pathway. This study seeks to provide new therapeutic strategies for SAKI‐induced inflammation, oxidative stress and mitochondrial damage.

## 2 MATERIALS AND METHODS

### 2.1 Cell culture and processing

Human renal tubular epithelial cells HK‐2 (SNL‐165) were obtained from Sunncell Biotechnology Co., Ltd., and were grown in special medium for human renal tubular epithelial cells (SNLM‐165, Sunncell Biotechnology). The medium was changed every 3 days, with a passaging ratio of 1:3, and the incubation temperature was 37°C containing 5% CO_2_ by volume.

Referring to Xu et al.,^28^ HK‐2 cells were exposed to LPS (100 μg/mL, HY‐D1056, MedChemExpress) for 24 h, in order to construct a model of HK‐2 cell injury. In the dose‐screening experiments, the HK‐2 cells in T‐CA treatment group was treated with 2.5, 5, 10, 20, 40, 80, and 160 μM of T‐CA (B1256523, BenchChem) for 24 h. For the following experiments, HK‐2 cells were treated with T‐CA (2.5, 5, 10, and 20 μM) for 2 h, followed by exposed to LPS (100 μg/mL) for 24 h. In the signaling pathway exploration, the HK‐2 cells in LPS + T‐CA + Phorbol 12‐myristate 13‐acetate (PMA) group were exposed to T‐CA (20 μM) for 2 h, subsequently treated with the NF‐κB activator PMA (1 μg/mL, HY‐18739, MedChemExpress) for 24 h,^29^ and finally exposed to LPS for 24 h. In the LPS + T‐CA + ML385 group, cells were exposed to T‐CA (20 μM) for 2 h, then treated with the Nrf2 inhibitor ML385 (20 μM, HY‐100523, MedChemExpress) for 24 h,^30^ and finally exposed to LPS for 24 h.

### 2.2 Cell transfection

GRK5 overexpression plasmid (GRK5) and its control (Vector) were provided from RiboBio Co., Ltd.. Control and experimental vectors were transfected into HK‐2 cells 30 min before T‐CA treatment according to the instructions for Lipofectamine 3000 (L3000001, Invitrogen). The cells were continuously cultured in the incubator for 48 h after transfection. Subsequently, Trizol reagent (15596026, Invitrogen) was used for extracting ribonucleic acid (RNA), and the transfection efficiency was reflected by detecting GRK5 level in HK‐2 cells.

### 2.3 Cell Counting Kit‐8 assay

HK‐2 cells were seed into 96‐well cell culture plates (1 × 10^4^ cells/well). After treatment with different concentrations of T‐CA, each well was supplemented with 10% Cell Counting Kit‐8 (CCK‐8) reagent (C0038, Beyotime). Cells were maintained at 37°C for 2 h, and then the OD_450_ values of the cells were measured by using a microplate reader (1410101, Thermo Fisher Scientific).

### 2.4 Lactate dehydrogenase assay

The lactate dehydrogenase (LDH) assay kit (C0016, Beyotime) was utilized to assess the cytotoxic effects on HK‐2 cells. HK‐2 cells were inoculated into 96‐well cell culture plates (1 × 10^4^ cells/well). After different treatments, the cells were centrifuged. Supernatant (120 μL) was collected, mixed well with LDH assay solution (60 μL), and incubated in the dark for 30 min. Then, the OD_490_ value was measured using a microplate reader to calculate the LDH release from the cells.

### 2.5 Superoxide dismutase, malondialdehyde, and glutathione measurement

The HK‐2 cell culture was aspirated and the cells were washed with pre‐cooled phosphate buffered saline (PBS). Superoxide dismutase (SOD) sample preparation solution from the SOD assay kit was added to lyse the cells. The lysate was centrifuged, and the supernatant was taken as the sample to be tested. The BCA protein assay kit (P0012, Beyotime) was utilized to assess the protein concentration, the protein concentration was quantified as 50 μg, mixed with SOD assay buffer and incubated for 30 min, and the OD_450_ value was measured. In addition, the above cell lysate supernatant was taken, and the levels of glutathione (GSH) and malondialdehyde (MDA) were measured following the protocols outlined in the MDA Assay Kit (S0131S, Beyotime) and GSH Assay Kit (S0053, Beyotime).

### 2.6 Hoechst 33258 staining

After different treatments, HK‐2 cells were seed into 12‐well plates and exposed to 4% paraformaldehyde (P0099, Beyotime) for 30 min. Added 500 μL of Hoechst 33258 staining solution (C1017, Beyotime) was added to adequately cover the cells. Following a 20‐min incubation period, the cells underwent two washes with PBS, after which their apoptotic status were examined using a fluorescence microscope (DM IL LED, Leica).

### 2.7 Mitochondrial superoxide staining

Mitochondrial reactive oxygen species (mROS) levels were assessed using a mitochondrial superoxide (MitoSOX) assay kit (S0061S, Beyotime). The 5 μM MitoSOX Red staining workup was prepared according to the kit instructions. HK‐2 cells were inoculated in 6‐well plates (1 × 10^6^ cells/well) and mixed well with 1 mL of MitoSOX Red staining solution. Following a 30‐min incubation at 37°C, cells were rinsed twice with PBS before examining the fluorescence intensity of mROS using a fluorescence microscope.

### 2.8 SAKI mouse model construction

Male C57BL/6 mice (25–30 g), aged 8 weeks, were acquired from Vitalriver and placed in a standard animal feeding room for 1 week of acclimatization. The rearing temperature was 22 ± 2°C, relative humidity 45%. All mice were allowed to eat and drink freely. Every effort was undertaken to limit the animal population and to lessen the pain caused to the animals in this study. The Henan Provincial People's Hospital Ethics Committee granted approval for the animal experiment.

Mice were divided equally into Sham, SAKI, SAKI + 10 mg/kg T‐CA, SAKI + 20 mg/kg T‐CA, SAKI + 40 mg/kg T‐CA, LPS + T‐CA + GRK5, LPS + T‐CA + Vector, SAKI + T‐CA + PMA, or SAKI + T‐CA + ML385 group in a random manner (*n* = 10). In SAKI + 10 mg/kg T‐CA, SAKI + 20 mg/kg T‐CA, and SAKI + 40 mg/kg T‐CA group, mice received intraperitoneal injections of 10, 20, and 40 mg/kg of T‐CA 2 h before CLP surgery, respectively.^31^, ^32^ T‐CA (40 mg/kg) was administered via intraperitoneal injection 2 h before CLP surgery, GRK5 or Vector was recombined into adenoviral vectors, and GRK5 or Vector was injected into the kidneys of mice 1 h after the surgery,^33^ which was recorded as the LPS + T‐CA + GRK5 group or LPS + T‐CA + Vector group. In addition, 40 mg/kg of T‐CA was administered 2 h before surgery and either PMA (5 mg/kg) or ML385 (30 mg/kg) was administered via intraperitoneal injection 1 h prior to surgery, which was recorded as SAKI + T‐CA + PMA group or SAKI + T‐CA + ML385 group. In the Sham group, only dissection was performed, but cecum ligation and perforation were not performed, and an equal amount of normal saline was injected. The SAKI group underwent the CLP operation and was injected with an equal amount of normal saline. We excluded the sample that died or failed the model establishment before endpoint. Ultimately, six mice were randomly selected from each group for analysis.

Referring to the method of Lin et al.,^34^ the SAKI model of mice was established using the CLP method. The mice were fasted for 12 h before surgery, received an intraperitoneal injection of 1.5% sodium pentobarbital (40 mg/kg) for anesthesia, shaved and disinfected with alcohol. A 15 mm long incision was performed in the center of the mouse abdomen to expose the subcutaneous connective tissue and open the muscular, fascial, and peritoneal layers layer by layer. The cecum was located on the left side of the mouse abdomen, and its distal end and the mesentery of the large intestine were carefully separated. At the terminal 1/4 of the cecum, a No. 4 intestinal thread was used to ligate the cecum, which was then punctured with 2 holes using an 18‐gauge needle. A small portion of the contents is extruded to ensure patency of the hole, and then the bowel is placed back into the abdominal cavity and the peritoneal fascial muscles and skin are sutured. Immediately after surgery, pre‐warmed 0.9% saline solution was administered for resuscitation. Twenty‐four hour after surgery, mice were executed using sodium pentobarbital (100 mg/kg) and blood and kidney tissues were gathered.

### 2.9 Serum creatinine and blood urea nitrogen levels measurement

Creatinine assay kit (C011‐2‐1) and urea nitrogen kit (C013‐2‐1) were acquired from the NanJing JianCheng Bioengineering Institute. The blood of mice from different treatment groups was left to stand for 1 h and the upper serum layer was collected. Serum creatinine (Scr) and blood urea nitrogen (BUN) levels in serum were determined according to the instructions.

### 2.10 Hematoxylin and eosin staining

Hematoxylin and eosin (HE) staining kit (C0105S, Beyotime) was used to assess renal tissue lesions in mice. Kidney tissues were exposed to 4% paraformaldehyde for 24 h, dehydrated and paraffin‐embedded in gradient ethanol (100%, 95%, 75%, and 50%) and sectioned (4 μm), and the slices were baked for 8 h at 65°C. The slices were deparaffinized in xylene (247642, Sigma‐Aldrich) and hydrated in gradient ethanol. Staining with hematoxylin staining solution for 8 min, differentiation solution (C0161s, Beyotime) for 30 s, and dying with eosin for 1 min. The sections were dehydrated in different concentrations of ethanol, xylene clear, and sealed with neutral gum (C0173, Beyotime). The samples were viewed through a microscope and the kidney tissue damage was scored on a total scale of 0–4, with higher scores representing more severe damage.^35^

### 2.11 Periodic acid‐schiff staining

Mouse kidney tissue damage was assessed by Periodic Acid‐Schiff (PAS) staining kit (C0142S, Beyotime). Paraffin blocks of mouse kidney tissue were routinely sectioned, deparaffinized with xylene and hydrated with gradient ethanol. Periodic acid solution (100 μL) was added dropwise on the surface of the samples, and the reaction was protected from light for 10 min, then washed using distilled water. Subsequently, Schiff reagent (100 μL) was added and left to incubate for 30 min at 37°C with light protection. The staining solution was removed, and then the samples were dyed with hematoxylin solution for 30 s. The samples were rinsed twice in distilled water, dehydrated in anhydrous ethanol for 2 min, cleared with xylene, observed and photographed under a microscope. Finally, kidney tissue damage was scored.^35^

### 2.12 TUNEL staining

Paraffin sections of mouse kidney tissue were deparaffinized with xylene and hydrated with gradient ethanol. DNase‐free proteinase K solution (20 μg/mL, ST532, Beyotime) was added and reacted for 30 min. Subsequently, Terminal Deoxynucleotidyl Transferase mediated dUTP Nick‐End Labeling (TUNEL) assay solution (C1086, Beyotime) was added dropwise and incubated in the dark for 1.5 h. The sections were then treated with 4’,6‐Diamidino‐2’‐phenylindole (DAPI) staining solution (D9542, Sigma‐Aldrich) and incubated away from light for 10 min. After sealing with AntiFade mounting medium (HY‐K1042, MedChemExpress), the slices were observed using a microscope and were photographed.

### 2.13 Measurement of ROS levels

Frozen sections of mouse kidney tissue made within 1 h of excision were spread with drops of cleaning solution over the entire surface of the sections and left to stand for 5 min. After shaking off the washing solution, 10 μM dihydroethidium probe (S0063, Beyotime) was added dropwise so that the probe evenly covered the tissue sections and left to incubate for 30 min in the darkness. Following two washes with PBS, cover slips were placed on the tissue sections, then examined and captured using a fluorescence microscope.

### 2.14 ELISA

To measure the secretion of inflammatory factors in HK‐2 cells, the Human Interleukin (IL)‐1β (PI305, Beyotime), IL‐6 (PI330, Beyotime), and tumor necrosis factor alpha (TNF‐α) ELISA Kit (PT518, Beyotime) were utilized. To assess the levels of oxidative stress markers in serum, mouse SOD (ml001998, Enzyme‐linked Biotechnology), GSH (ml063305, Enzyme‐linked Biotechnology), and MDA ELISA Kit (S18649, Sailuofei Biotechnology) were utilized. HK‐2 cell culture supernatant or mouse serum was introduced into ELISA well plates and allowed to incubate for 2 h. After adding PBS to wash for 3 times, each well plate received the corresponding antibody (100 μL), which was then incubated for 1 h. The well plates were rinsed 3 times with washing buffer, the solution was shaken off, and the sample was then incubated with streptavidin‐HRP for half an hour. Then the color developer 3,3',5,5'‐Tetramethylbenzidine (TMB) solution was added and left to incubate for 10 min, 50 μL of termination solution was introduced and mixed well, and the OD_450_ value was measured.

### 2.15 Immunofluorescence

HK‐2 cells after different treatments were inoculated in 12‐well plates. Once the cell density reached 50%–60%, the cells were fixed with 4% paraformaldehyde for 20 min. Paraffin sections of mouse kidney tissue were deparaffinized, hydrated with gradient ethanol, and then antigen repaired. Cell surfaces or tissue sections were permeabilized with drops of 0.3% Tritonx‐100 (X100, Sigma‐Aldrich) for 10 min 5% bovine serum albumin (BSA, V900933, Sigma‐Aldrich) was added for closure for 30 min. NF‐κB primary antibody (14‐6731‐81, 1:100, Invitrogen) was added dropwise on the cell surface, and F4/80 primary antibody (MA5‐16363, 1:100, Invitrogen) was added dropwise on the surface of the tissue sections, and incubated overnight at 4°C. The next day, the tissue sections were incubated with FITC‐labeled goat anti‐rabbit IgG (F‐2765, 1:100, Invitrogen) for 1 h at 37°C away from light, and then they were exposed to DAPI stain (C1005, Beyotime) for 10 min, and observed using fluorescence microscopy. Fluorescence intensity of NF‐κB and F4/80 was analyzed with Image J software (version 1.54 h, Wavne Resband, National Institute of Mental Health).

### 2.16 Mitochondrial membrane potential (MMP) assay

The mitochondrial membrane potential (MMP) assay kit (C2006, Beyotime) was utilized to assess alterations in MMP levels within HK‐2 cells and kidney tissues from mice. HK‐2 cells (1 × 10^5^) were resuspended in cell culture medium, and mixed well with JC‐1 staining solution, and reacted for 20 min at 37°C. After centrifugation, the cell precipitate was taken and rinsed two times with JC‐1 staining buffer, centrifuged again, the precipitated cells were taken and resuspended in the same buffer, and observed by a fluorescence microscope. Mitochondria were extracted and purified from mouse kidney tissue utilizing the tissue mitochondrial isolation kit (C3606, Beyotime), and a JC‐1 staining working solution (0.9 mL) was mixed well with purified mitochondria (0.1 mL), and then directly observed by fluorescence microscopy.

### 2.17 Detection of ultrastructure

Kidney tissues from mice were collected and treated with 2.5% glutaraldehyde (G6257, Sigma‐Aldrich) at 4°C overnight. Following two rinses with PBS, the samples were exposed to 1% osmium acid (O5500, Sigma‐Aldrich) for 2 h. Gradient ethanol (30%, 50%, 70%, 80%, 95%, and 100%) dehydration was conducted for 10 min each time, with a final dehydration step using 100% ethanol. Next, dehydration was performed twice with 100% acetone for 10 min every time. After dehydration, they were embedded and maintained at 60°C for 48 h of polymerization. Samples were thinly sliced to 60 nm thickness and subjected to a double‐staining and dried after copper mesh fishing. The samples were then observed using a transmission electron microscope (TEM, S4800, Hitachi).

### 2.18 Adenosine 5′‐triphosphate production assay

Adenosine 5′‐triphosphate (ATP) production in HK‐2 cells and mouse kidney tissues was detected through an ATP assay kit (S0026, Beyotime). Cells were inoculated in a 6‐well plate, mixed well with lysis solution (200 μL) to fully lyse the cells, then centrifuged, and the supernatant was taken as a backup. Mouse kidney tissue (20 mg) was weighed, lysis solution (200 μL) was added, fully homogenized, and then centrifuged for 5 min. 20 μL of sample was taken, quickly mixed with ATP assay working solution (100 μL), and chemiluminescence was detected using a tecan spark multifunctional enzyme labeling instrument (Tecan Trading AG).

### 2.19 Western blot

Radio Immunoprecipitation Assay (RIPA) lysis buffer (P0013B, Beyotime) was added to different treated cells or mouse kidney tissues and lysed sufficiently to obtain proteins, and detected the contents by BCA protein assay kit. Next, proteins were isolated using sodium dodecyl sulfate ‐ polyacrylamide gel electrophoresis (SDS‐PAGE) gels (12%, Invitrogen), and then they were shifted to polyvinylidene difluoride membranes (Invitrogen) and incubated with 5% BSA for 3 h. After rinsing the membranes, they were placed at 4°C for an overnight incubation with NF‐κB p65 primary antibody (14‐6731‐81, 1:1000, Invitrogen), caspase‐3 primary antibody (700182, 1:1000, Invitrogen), Cleaved caspase‐3 primary antibody (ab32024, 1:500, Abcam), Bax primary antibody (ab53154, 1:1000, Abcam), Bcl‐2 primary antibody (ab59348, 1:500, Abcam), p‐NF‐κB p65 primary antibody (MA5‐15160, 1:1000, Invitrogen), GRK5 primary antibody (PA5‐106484, 1:1000, Invitrogen), Nrf2 primary antibody (PA5‐27882, 1:1000, Invitrogen), heme oxygenase‐1 (HO‐1) primary antibody (PA5‐77833, 1:100, Invitrogen), dynamin‐related protein 1 primary antibody (DRP‐1, ab184247, 1:1000, Abcam), mitofusin 1 primary antibody (MFN‐1, ab221661, 1:1000, Abcam), or Optic Atrophy 1 primary antibody (OPA‐1, ab157457, 1:1000, Abcam). On the second day, followed by being rinsed thrice, the membranes were exposed to goat anti‐rabbit secondary IgG (31460, 1:10,000, Invitrogen) for 2 h. The chemiluminescent agent enhanced chemiluminescence (ECL, HY‐K1005, MedChemExpress) was evenly dripped onto the membrane, and the membrane was scanned with a gel imaging system (iBright CL1500, Invitrogen). Using Image J software to analyze gray value, and the relative expression was expressed as the ratio of its gray value to that of glyceraldehyde ‐3‐phosphate dehydrogenase (GAPDH, PA1‐987, 1:1000, Invitrogen).

### 2.20 Data processing and analysis

Every experiment was conducted a minimum of three times, and the outcomes are presented as mean ± standard deviation. For statistical analysis of data, we employed the SPSS 26.0 software (IBM SPSS Statistics 26). The *t*‐test was employed for group comparisons when the data met the criteria of normal distribution and homogeneity of variance; in instances where the data followed a normal distribution but exhibited not homogeneous variance, the corrected *t*‐test was used, whereas when the data did not follow a normal distribution, the nonparametric Wilcoxon rank sum test was used. The analysis of differences between multiple groups was conducted through one‐way Analysis of Variance (ANOVA), and two‐by‐two comparisons were made using the Least Significant Difference (LSD) method when the results showed a difference. *p* < 0.05 represents a significant difference. Plotting was done using Prism software (Graphpad 9.0).

## 3 RESULTS

### 3.1 T‐CA protects HK‐2 cells from LPS‐caused active damage

**FIGURE 1:**
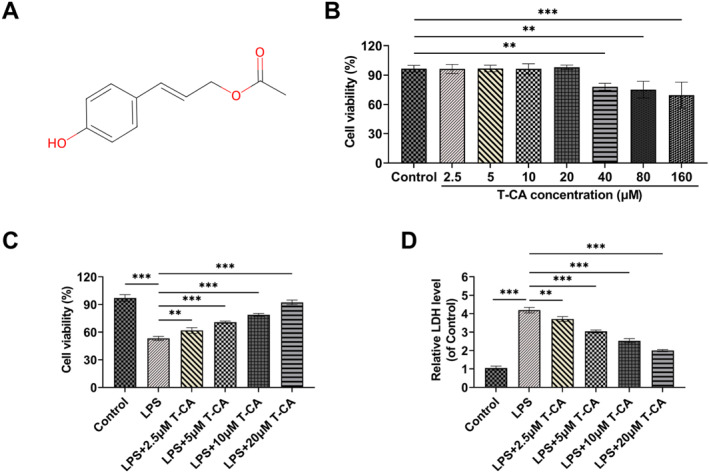
T‐CA ameliorates LPS‐induced HK‐2 cell damage. (A) Chemical structural formula of T‐CA. (B) The viability of HK‐2 cells was assessed by CCK8 assay following exposure to various concentrations of T‐CA (2.5, 5, 10, 20, 40, 80, 160 μM) in order to screen for suitable treatment concentrations. (C) CCK8 assay to detect the protective impacts of T‐CA (2.5, 5, 10, 20 μM) against LPS‐induced injury. (D) LDH Kit detected LDH release in HK‐2 cells to assess cytotoxicity. All data are presented as mean ± standard deviation, with experiments repeated in triplicate (*n* = 3 biological replicates) (**p < 0.01, ***p < 0.001). LDH, lactate dehydrogenase; LPS, lipopolysaccharide; T‐CA, Trans‐Coumaryl acetate.

The chemical structural formula of T‐CA is shown in Figure 1A. The viability of HK‐2 cells after 24 h of exposure to T‐CA was assessed using CCK‐8 assay to screen for the appropriate treatment concentrations. The results showed that treatment with 2.5, 5, 10, and 20 μM of T‐CA did not lead to any notable alterations in the viability of HK‐2 cells. When the concentration was raised to 40–160 μM, HK‐2 cell activity was significantly reduced, suggesting that the high concentration of T‐CA (40–160 μM) had a certain toxicity effect on HK‐2 cells (Figure 1B), and therefore, T‐CA at 2.5–20 μM was selected for subsequent experiments. After 100 μg/mL LPS treatment, HK‐2 cell viability was notably reduced, but 2.5–20 μM T‐CA all significantly increased cell viability, and the higher the concentration the better the effect (Figure 1C). In addition, LDH release is commonly used to assess cellular damage. LDH release from HK‐2 cells was markedly elevated following LPS treatment, and T‐CA treatment significantly reduced LDH release, and the higher the concentration of T‐CA, the lower the amount of LDH release (Figure 1D).

### 3.2 T‐CA ameliorates inflammation and oxidative stress caused by LPS and reduces apoptosis and mitochondrial damage in HK‐2 cells

**FIGURE 2:**
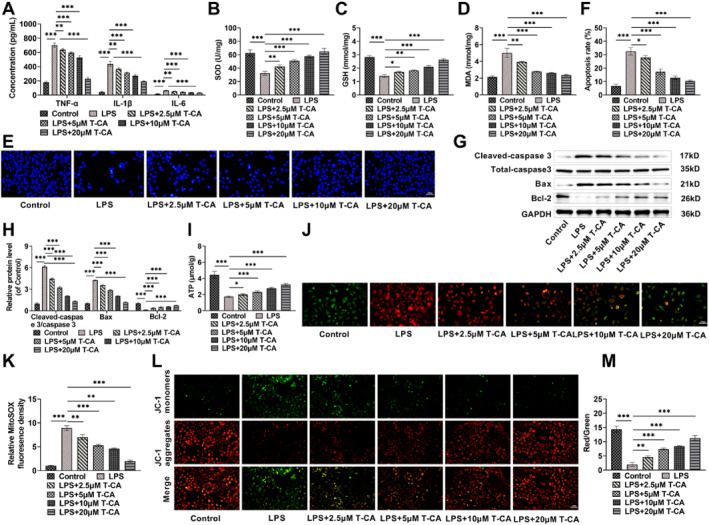
T‐CA ameliorates lipopolysaccharide‐caused inflammation and oxidative stress in HK‐2 cells. (A) The ELISA kit results showed that T‐CA declined tumor necrosis factor alpha, IL‐1β, and IL‐6 levels. (B–D) T‐CA increased Superoxide dismutase and glutathione levels and decreased malondialdehyde levels in HK‐2 cells. (E,F) Hoechst 33258 staining confirmed that T‐CA reduced apoptosis (20×, 100 μm). (G,H) Western blot measured that T‐CA treatment decreased Cleaved‐caspase‐3/Caspase 3 and Bax levels and increased the Bcl‐2 level. (I) T‐CA increased ATP levels as measured by the ATP kit. (J,K) MitoSOX staining to detect mitochondrial ROS (mROS) fluorescence abundance confirmed that T‐CA reduced mROS levels (40×, 50 μm). (L,M) JC‐1 fluorescence detected mitochondrial membrane potential (MMP), which produces red fluorescence in normal cells and green fluorescence when the MMP is lowered (20×, 100 μm). All data are presented as mean ± standard deviation, with experiments repeated in triplicate (*n* = 3 biological replicates) (*p < 0.05, **p < 0.01, ***p < 0.001). ATP, adenosine 5′‐triphosphate; T‐CA, Trans‐Coumaryl acetate.

Using ELISA kits, we found that LPS treatment caused a notable rise in TNF‐α, IL‐1β, and IL‐6 levels in HK‐2 cells, but T‐CA treatment reduced the inflammatory effects of LPS, leading to a marked decline in these inflammatory factors levels, and the higher the concentration of T‐CA, the better the anti‐inflammatory effects (Figure 2A). Furthermore, LPS markedly declined the content of antioxidant enzyme SOD and antioxidant GSH, and elevated the content of lipid peroxidation marker MDA in HK‐2 cells, whereas T‐CA treatment suppressed LPS‐induced oxidative stress (Figure 2B–D). By Hoechst 33258 staining, following LPS treatment, it could be observed that nuclear condensation and a large amount of cellular debris appeared in HK‐2 cells, and the apoptosis rate was notably elevated, and T‐CA treatment attenuated LPS‐induced cellular damage and alleviated apoptosis (Figure 2E,F). Western blot findings indicated that LPS caused a notable increase in apoptosis marker Cleaved‐caspase‐3/Caspase 3 and Bax levels and a significant reduction in anti‐apoptotic protein Bcl‐2, while T‐CA declined the Cleaved‐caspase‐3/Caspase 3 and Bax levels and elevated Bcl‐2 level, which indicated that T‐CA inhibited apoptosis caused by LPS, which aligns with the findings from Hoechst 33258 staining (Figure 2G,H). Notably, LPS significantly decreased ATP production in HK‐2 cells, whereas T‐CA treatment increased ATP levels, suggesting that T‐CA alleviated LPS‐induced mitochondrial damage (Figure 2I). MitoSOX staining is commonly used to detect mROS production and accumulation, and LPS resulted in significantly elevated mROS levels in HK‐2 cells, whereas T‐CA inhibited excessive mROS accumulation (Figure 2J,K). The JC‐1 fluorescent probe is widely used to assess MMP. After treatment with LPS, HK‐2 cells exhibited a strong green fluorescence, indicating that MMP was reduced, whereas T‐CA treatment led to an increase in red fluorescence intensity, and the higher the concentration, the higher the intensity of red fluorescence, further confirming that T‐CA could alleviate mitochondrial damage (Figure 2L,M). In view of the concentration‐dependent protective effect of T‐CA on HK‐2 cells, we used 20 μM T‐CA to treat HK‐2 cells in subsequent experiments.

### 3.3 T‐CA suppresses NF‐κB activation and induces Nrf2 signaling pathway activation

**FIGURE 3:**
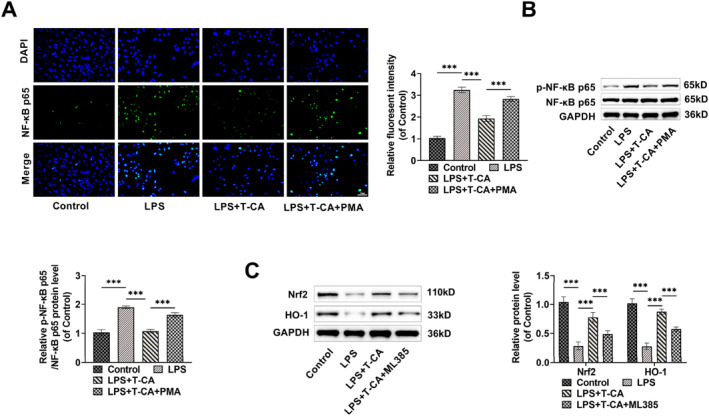
T‐CA suppresses NF‐κB activation and induces Nrf2 pathway activation (A) Immunofluorescence detection of the impact of T‐CA on NF‐κB nuclear translocation (40×, 50 μm). (B,C) Western blot measured that T‐CA decreased p‐NF‐κB p65/NF‐κB p65 levels and increased Nrf2 and HO‐1 levels in HK‐2 cells. All data are presented as mean ± standard deviation, with experiments repeated in triplicate (*n* = 3 biological replicates) (***p < 0.001). Nrf2, nuclear factor erythroid‐2 related factor 2; T‐CA, Trans‐Coumaryl acetate.

In order to explore how T‐CA protects HK‐2 cells, we utilized immunofluorescence staining to monitor the translocation of NF‐κB into the nucleus. LPS treatment caused activated NF‐κB to translocate into the nucleus, whereas T‐CA inhibited this nuclear translocation, and treatment with the NF‐κB activator PMA attenuated the effect of T‐CA (Figure 3A). Additionally, LPS treatment promoted NF‐κB phosphorylation, while T‐CA inhibited it, and PMA weakened the inhibitory impact of T‐CA on NF‐κB phosphorylation, which indicates that T‐CA may exert a protective impact on HK‐2 cells by inhibiting NF‐κB activation (Figure 3B). Then, we further explored the effect of T‐CA on Nrf2 pathway. Nrf2 and HO‐1 levels were notably reduced by LPS treatment, whereas T‐CA elevated the expression of these two proteins, and the Nrf2 inhibitor ML385 attenuated the effect of T‐CA (Figure 3C). The above results findings indicate T‐CA may exert a protective impact on HK‐2 cells by hindering NF‐κB activation and activating Nrf2 pathway.

### 3.4 T‐CA mediates the NF‐κB/Nrf2 pathway through GRK5 and ameliorates LPS‐caused HK‐2 cell injury

**FIGURE 4:**
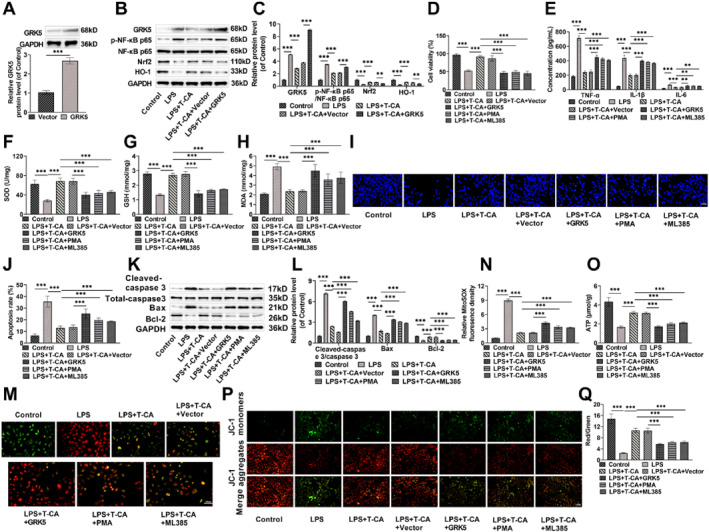
T‐CA mediates the NF‐κB/Nrf2 pathway through GRK5 and ameliorates lipopolysaccharide‐induced injury in HK‐2 cells. (A) Vector or GRK5 was transfected in HK‐2 cells, and Western blot measured that GRK5 level was increased after transfection, indicating successful transfection. (B,C) Western blot was performed to assess GRK5, p‐NF‐κB p65/NF‐κB p65, Nrf2 and HO‐1 levels. (D) CCK8 assay measured that T‐CA (20 μM) treatment increased the cell viability, while PMA, ML385 treatment or overexpression of GRK5 resulted in decreased cell viability. (E) T‐CA decreased tumor necrosis factor alpha, IL‐1β, and IL‐6 levels, but PMA, ML385 treatment, or overexpression of GRK5 elevated the inflammatory factors levels as measured by ELISA kit. (F–H) PMA, ML385 treatment, or overexpression of GRK all resulted in decreased superoxide dismutase and glutathione levels and increased malondialdehyde levels. (I,J) Hoechst 33258 staining confirmed that PMA, ML385 treatment or overexpression of GRK increased apoptosis (20×, 100 μm). (K,L) Cleaved‐caspase 3/Caspase 3 and Bax levels were elevated, with Bcl‐2 was decreased after PMA, ML385 treatment or overexpression GRK, as measured via Western blot. (M,N) MitoSOX staining to detect mitochondrial ROS (mROS) fluorescence abundance, and PMA, ML385 treatment or overexpression of GRK resulted in elevated mROS levels (40×, 50 μm). (O) ATP levels in HK‐2 cells were reduced by PMA, ML385 treatment or overexpression of GRK as measured by the ATP kit. (P,Q) JC‐1 fluorescence detected mitochondrial membrane potential in HK‐2 cells (20×, 100 μm). All data are presented as mean ± standard deviation, with experiments repeated in triplicate (*n* = 3 biological replicates) (**p < 0.01, ***p < 0.001). ATP, adenosine 5′‐triphosphate; GRK5, G protein‐coupled receptor kinase 5; Nrf2, nuclear factor erythroid‐2 related factor 2; PMA, Phorbol 12‐myristate 13‐acetate; T‐CA, Trans‐Coumaryl acetate.

Based on the results of the pre‐experiment, we found that T‐CA could bind to GRK5, so we hypothesized that T‐CA might mediate the NF‐κB/Nrf2 pathway by regulating GRK5, thus exerting a protective effect on HK‐2 cells. We transfected Vector/GRK5 into HK‐2 cells and verified its transfection efficiency by Western blot. After transfection of GRK5, GRK5 level was markedly elevated, which indicated that the transfection was successful and subsequent functional experiments could be performed (Figure 4A). Notably, LPS treatment increased GRK5 expression, whereas T‐CA decreased GRK5 expression; the transfection of GRK5 impaired the down‐regulation of GRK5 by T‐CA. Furthermore, overexpression of GRK5 elevated p‐NF‐κB level and decreased Nrf2 and HO‐1 levels, suggesting that GRK5 regulates the NF‐κB/Nrf2 pathway (Figure 4B,C). Overexpression of GRK5 significantly reduced the protective effect of T‐CA on HK‐2 cell viability, whereas the NF‐κB activator PMA and the Nrf2 inhibitor ML385 had the same effect (Figure 4D). In addition, PMA, ML385 treatment and overexpression of GRK5 all reduced the inhibit impact of T‐CA on the secretion of inflammatory factors, and decreased the content of SOD and GSH and increased MDA level (Figure 4E–H). Moreover, PMA, ML385 treatment and overexpression of GRK5 also increased apoptosis rate, resulting in significantly higher Cleaved‐caspase‐3/Caspase 3 and Bax levels, and markedly higher Bcl‐2 level (Figure 4I–L). PMA, ML385 treatment, and overexpression of GRK5 all increased the fluorescence intensity of mROS, inhibited ATP production, and decreased MMP (Figure 4M–Q). These results indicate that overexpression of GRK5 modulates the NF‐κB/Nrf2 pathway and impairs the protective role of T‐CA in preventing damage to HK‐2 cell, suggesting that T‐CA modulates the NF‐κB/Nrf2 pathway through GRK5, which in turn protects HK‐2 cells.

### 3.5 T‐CA attenuates kidney injury in SAKI mice

**FIGURE 5:**
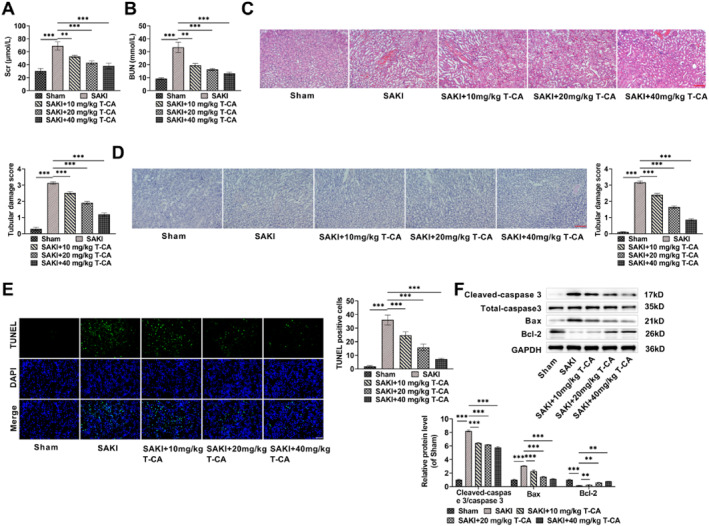
T‐CA attenuates kidney injury in SAKI mice. (A,B) T‐CA declined Scr and blood urea nitrogen levels in SAKI mice as measured by creatinine assay kit and urea nitrogen kit. (C) Hematoxylin and eosin staining showed that T‐CA treatment alleviated vacuolization of renal tubular cells and reduced tubular injury (20×, 100 μm). (D) Periodic acid‐schiff staining results confirmed that T‐CA treatment improved renal tubular injury in SAKI mice (20×, 100 μm). (E) TUNEL staining confirmed that T‐CA reduced apoptosis in renal tissues of SAKI mice (40×, 50 μm). (F) Western blot measured that T‐CA treatment decreased Cleaved‐caspase 3/caspase 3, Bax levels and increased Bcl‐2 protein level in renal tissues. All data are presented as mean ± standard deviation, 6 mice per group (*n* = 6) (**p < 0.01, ***p < 0.001). SAKI, septic acute kidney injury; T‐CA, Trans‐Coumaryl acetate; TUNEL, Terminal Deoxynucleotidyl Transferase mediated dUTP Nick‐End Labeling.

Scr and BUN are important indicators for assessing renal impairment, and elevated levels of Scr and BUN indicate the presence of renal insufficiency with reduced glomerular filtration rate.^36^ Therefore, we examined the effects of different doses of T‐CA treatment on Scr and BUN levels in SAKI mice. Compared to the Sham group, the SAKI group of mice showed a notable rise in Scr and BUN levels, indicating that the glomerular filtration rate of mice was reduced. Notably, Scr and BUN levels were declined after T‐CA treatment, and the effect of T‐CA treatment was dose‐dependent, confirming that T‐CA improved glomerular filtration rate (Figure 5A,B). Next, we observed the pathological alterations in kidney tissues by HE staining and PAS staining. The results showed that SAKI mice developed significant renal histomorphometric abnormalities, as evidenced by vacuolization of renal tubular cells, and significantly higher kidney injury scores. T‐CA treatment dose‐dependently alleviated renal tissue injury and reduced renal injury scores in SAKI mice (Figure 5C,D). Apoptosis in the kidney is a prominent feature of the pathogenesis of SAKI, and we examined apoptosis in renal tissues by TUNEL staining and Western blot. The amount of TUNEL‐positive cells in renal tissues of SAKI mice was significantly higher, as well as Bax and Cleaved‐caspase 3/caspase 3 levels, with Bcl‐2 level was markedly lower, confirming that SAKI could lead to apoptosis in renal tissues of mice. T‐CA treatment markedly declined the amount of TUNEL‐positive cells and apoptosis‐related protein levels, and increased Bcl‐2 level, suggesting that T‐CA attenuates renal tissue injury in SAKI mice (Figure 5E,F).

### 3.6 T‐CA attenuates kidney injury in SAKI mice through GRK5‐mediated NF‐κB/Nrf2 pathway

**FIGURE 6:**
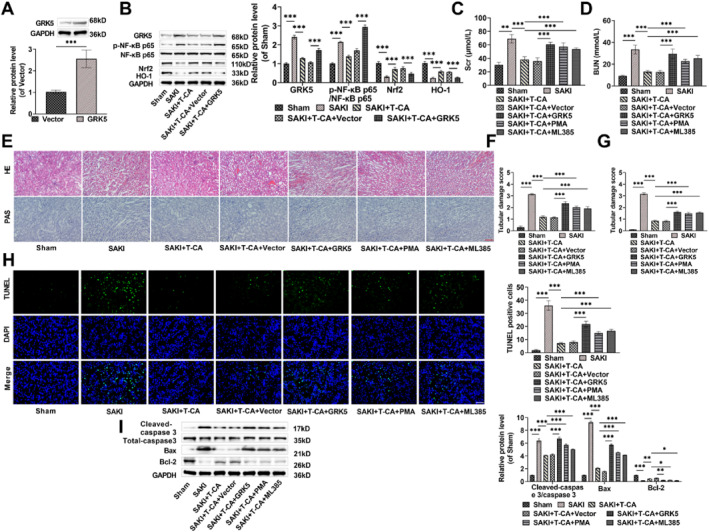
T‐CA attenuates renal injury in SAKI mice by GRK5‐mediated NF‐κB/Nrf2 pathway. (A) GRK5 was successfully overexpressed in renal tissues, as examined by Western blot. (B) Western blot detection of GRK5, p‐NF‐κB p65/NF‐κB p65, Nrf2, and HO‐1 protein levels in renal tissues. (C,D) PMA, ML385 treatment, or overexpression of GRK5 decreased Scr and blood urea nitrogen levels in SAKI mice as measured by the creatinine assay kit and the urea nitrogen kit. (E–G) Hematoxylin and eosin staining (E upper) and periodic acid‐schiff staining (E lower) were used to assess tubular injury in renal tissues of SAKI mice (20×, 100 μm). (H) TUNEL staining confirmed that PMA, ML385 treatment or overexpression of GRK5 promoted apoptosis in renal tissue cells (40×, 50 μm). (I) PMA, ML385 treatment or overexpression of GRK5 resulted in increased Cleaved‐caspase 3/caspase 3 and Bax levels and decreased Bcl‐2 protein level in kidney tissues as assessed through Western blot. All data are presented as mean ± standard deviation, 6 mice per group (*n* = 6) (*p < 0.05, **p < 0.01, ***p < 0.001). GRK5, G protein‐coupled receptor kinase 5; Nrf2, nuclear factor erythroid‐2 related factor 2; PMA, Phorbol 12‐myristate 13‐acetate; SAKI, septic acute kidney injury; T‐CA, Trans‐Coumaryl acetate; TUNEL, Terminal Deoxynucleotidyl Transferase mediated dUTP Nick‐End Labeling.

Referring to the method of Gao et al.,^33^ we injected Vector/GRK5 into mouse kidneys and examined GRK5 level through Western blot, revealing a notably elevated level of GRK5 in mouse kidney tissues after injection of GRK5, which allowed for subsequent experiments (Figure 6A). Consistent with the results of cellular experiments, T‐CA treatment reduced GRK5 level in kidney tissues, and also declined NF‐κB phosphorylation level and elevated Nrf2 and HO‐1 expression. In contrast, overexpression GRK5 increased NF‐κB phosphorylation and decreased Nrf2 and HO‐1 levels (Figure 6B). Additonally, overexpression of GRK5 attenuated the therapeutic effect of T‐CA, resulting in significantly higher Scr and BUN levels, renal tubular injury scores, and TUNEL‐positive cells in mice, whereas injection of the NF‐κB activator PMA or the Nrf2 inhibitor ML385 also had a similar effect to overexpression of GRK5 (Figure 6C–H). In addition, PMA, ML385 treatment and overexpression of GRK5 all notably elevated Cleaved‐caspase 3/caspase 3 and Bax levels, and downregulated Bcl‐2 (Figure 6I). In summary, overexpression of GRK5 was able to further aggravate renal injury in SAKI mice, confirming that T‐CA may ameliorate renal injury by regulating the expression of GRK5; meanwhile, overexpression GRK5 regulated the NF‐κB/Nrf2 pathway, whereas T‐CA hindered NF‐κB activation and activated the Nrf2 pathway; therefore, we hypothesized that T‐CA attenuated renal injury in SAKI mice through GRK5‐mediated NF‐κB/Nrf2 pathway.

### 3.7 T‐CA attenuates renal oxidative stress and inflammatory responses in SAKI mice by GRK5/NF‐κB/Nrf2 pathway

**FIGURE 7:**
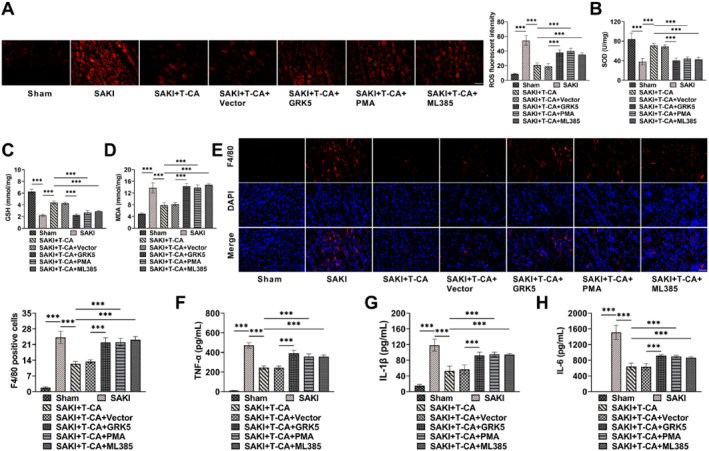
T‐CA attenuates renal oxidative stress and inflammatory responses in SAKI mice through GRK5/NF‐κB/Nrf2 signaling pathway. (A) Dihydroethidium staining results confirmed that T‐CA treatment reduced ROS levels in renal tissues, but PMA, ML385 treatment or overexpression of GRK5 resulted in increased ROS levels (20×, 100 μm). (B–D) T‐CA treatment increased superoxide dismutase and glutathione levels and declined malondialdehyde level in SAKI mice serum, but PMA, ML385 treatment or overexpression of GRK5 attenuated the therapeutic effect of T‐CA. (E) Immunofluorescence staining results confirmed that T‐CA treatment reduced F4/80 expression in renal tissues, but PMA, ML385 treatment or overexpression of GRK5 attenuated the therapeutic effect of T‐CA (40×, 50 μm). (F–H) T‐CA treatment reduced tumor necrosis factor alpha, IL‐1β, and IL‐6 levels as measured by ELISA kits, but PMA, ML385 treatment, or overexpression GRK5 raised inflammatory factors levels. All data are presented as mean ± standard deviation, 6 mice per group (*n* = 6) (***p < 0.001). GRK5, G protein‐coupled receptor kinase 5; Nrf2, nuclear factor erythroid‐2 related factor 2; PMA, Phorbol 12‐myristate 13‐acetate; SAKI, septic acute kidney injury; T‐CA, Trans‐Coumaryl acetate.

Next, we explored whether T‐CA attenuated renal oxidative stress and inflammation in SAKI mice by modulating GRK5/NF‐κB/Nrf2 pathway. T‐CA markedly reduced ROS and MDA levels and elevated the contents of SOD and GSH in SAKI mice, whereas PMA, ML385 treatment, and overexpression of GRK5 attenuated the therapeutic effect of T‐CA, resulting in elevated ROS and MDA levels, and decreased contents of SOD and GSH (Figure 7A–D). The kidneys of SAKI mice exhibited heightened F4/80 expression, indicating the presence of macrophage infiltration; the number of these infiltrated macrophages was markedly reduced after injection of T‐CA, whereas the inhibitory impact of T‐CA on F4/80 expression was attenuated by PMA, ML385 treatment and overexpression of GRK5 (Figure 7E). The TNF‐α, IL‐1β, and IL‐6 levels were notably elevated in SAKI mice serum, but T‐CA treatment suppressed the secretion of inflammatory factors, whereas PMA, ML385 treatment, and overexpression of GRK5 all attenuated the therapeutic effect of T‐CA (Figure 7F–H). The above results indicated that PMA, ML385 treatment and overexpression of GRK5 aggravated kidney oxidative stress and inflammatory responses in mice, suggesting that T‐CA attenuated renal injury in SAKI mice by GRK5/NF‐κB/Nrf2 pathway.

### 3.8 T‐CA ameliorates renal mitochondrial dysfunction in SAKI mice via the GRK5/NF‐κB/Nrf2 signaling pathway

**FIGURE 8:**
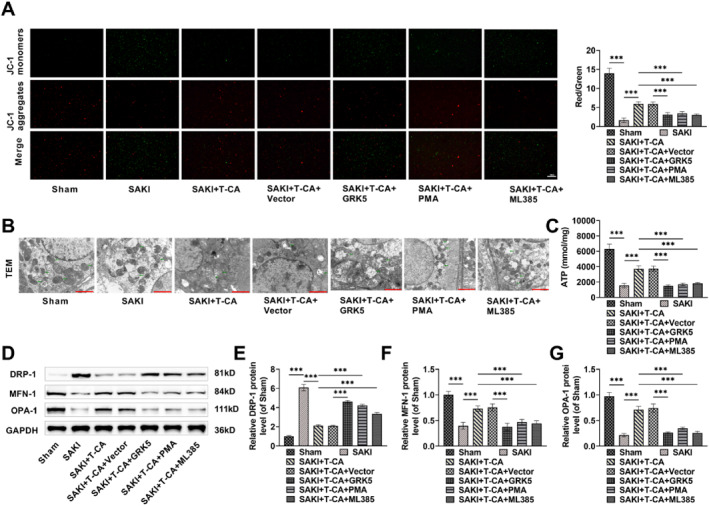
T‐CA ameliorates renal mitochondrial dysfunction in septic acute kidney injury mice by GRK5/NF‐κB/Nrf2 signaling pathway. (A) JC‐1 fluorescence detection of mitochondrial membrane potential in renal tissue, which produces red fluorescence in normal cells and green fluorescence when the membrane potential is lowered (40×, 50 μm). (B) TEM was used to detect mitochondrial morphology in proximal renal tubule cells of the kidney (15k×, 2 μm). (C) ATP kit to detect renal tissues and assess the ATP production capacity of mouse kidney. (D–G) Western blot measured that T‐CA treatment decreased DRP‐1 and MFN‐1 levels and increased OPA‐1 level in mouse kidney tissues, but PMA, ML385 treatment or overexpression of GRK5 could weaken the therapeutic effect of T‐CA. All data are presented as mean ± standard deviation, 6 mice per group (*n* = 6) (***p < 0.001). ATP, adenosine 5′‐triphosphate; GRK5, G protein‐coupled receptor kinase 5; Nrf2, nuclear factor erythroid‐2 related factor 2; PMA, Phorbol 12‐myristate 13‐acetate; T‐CA, Trans‐Coumaryl acetate; TEM, transmission electron microscope.

Dysfunctional mitochondria are a crucial contributor to the pathogenesis of SAKI^28^, ^37^; therefore, we explored the effect of T‐CA on renal mitochondrial dysfunction in SAKI mice. SAKI mice showed green fluorescence in kidney tissues, indicating reduced MMP, whereas injecting T‐CA resulted in increased intensity of red fluorescence, and PMA, ML385 treatment and overexpression of GRK5 attenuated the effect of T‐CA (Figure 8A). Transmission electron microscopy images showed pathologically altered mitochondrial structure in renal tubular cells of SAKI mice, characterized by rounded and smaller tubular mitochondria; missing, fragmented, and swollen mitochondrial cristae; and the appearance of tiny vacuoles in the mitochondrial matrix. In contrast, mitochondrial damage was significantly reduced in renal tubular cells in the SAKI + T‐CA group, and the mitochondrial cristae were more intact. However, the SAKI + T‐CA + GRK5, SAKI + T‐CA + PMA, and SAKI + T‐CA + ML385 groups aggravated the mitochondrial damage in renal tubular cells in the SAKI + T‐CA group (Figure 8B). In addition, the renal tissues of SAKI mice showed diminished ATP production, markedly elevated DRP‐1 (mitochondrial fission protein) level, and notably reduced MFN‐1 and OPA‐1 (mitochondrial fusion proteins) levels, confirming that mitochondria in renal tissues of SAKI mice were dysfunctional (Figure 8C–G). Although injection of T‐CA increased the ATP production capacity of renal tissues and reversed the aberrant expression of the above mentioned mitochondria‐related proteins, PMA, ML385 treatment and overexpression of GRK5 reduced the therapeutic effect of T‐CA. The above results further suggest that T‐CA attenuates mitochondrial dysfunction in SAKI mice by the GRK5/NF‐κB/Nrf2 pathway.

## 4 DISCUSSION

Previous studies have reported that LPS is an ideal drug for inducing experimental sepsis, and LPS‐induced HK‐2 cell model and CLP‐constructed animal model of SAKI have been widely used in the study of SAKI.^38^, ^39^ We constructed a HK‐2 cell injury model and a SAKI mouse model with reference to the above methods. This research indicated that T‐CA treatment could attenuate LPS‐induced HK‐2 cell injury, and it could improve renal pathological injury, reduce Scr and BUN levels, and improve renal function of SAKI mice after intraperitoneal injection of T‐CA in the mice, implying that T‐CA might serve as a potential therapeutic option for SAKI.

During the development of sepsis, the uncontrollable inflammatory response can cause acute functional damage in several organs of the body, and the kidneys being among the most frequently attacked organs by the inflammatory response.^40^ Several studies have demonstrated that inflammation is a key process and major contributor to renal impairment in SAKI.^37^, ^41^ Various inflammatory cytokines in the blood of SAKI patients can induce the generation of intravascular microthrombi and cause the apoptosis of significant quantity of lymphocytes, which reduces intrarenal blood flow and further aggravates inflammatory injury.^42^ Reducing the inflammatory response causes a decrease in macrophage presence in cases of acute kidney injury thereby reducing organ injury and dysfunction during sepsis.^43^ Therefore, one of the focuses of assessing the effectiveness of pharmacologic interventions for SAKI is whether they reduce inflammatory damage. Daphnetin, also a natural coumarin derivative, has been found to diminish the release of pro‐inflammatory cytokines in the serum of eptic lung injury mice and to attenuate lung pathology.^17^ Our study revealed that LPS promoted the release of inflammatory factors in HK‐2 cells, which were also present in large amounts in the serum of SAKI mice. After treatment with T‐CA, the inflammatory factor levels in HK‐2 cells and serum of SAKI mice were declined and renal histopathological changes were attenuated, demonstrating that T‐CA can provide a protective role for kidney tissues in SAKI by reducing inflammatory factor levels.

In addition to inflammatory injury, oxidative stress also is crucial in SAKI. Oxidative stress directly induces the production of ROS, and the accumulation of ROS in renal tissues can lead to structural damage and dysfunction of renal tissues.^44^ Evidence suggests that inhibiting indicators of oxidative stress and lowering pro‐inflammatory factors levels can alleviate apoptosis and inflammatory responses in the SAKI model.^45^ Esculin is also one of the coumarin derivatives that has been reported to alleviate cytokine release and attenuate oxidative stress‐related injury in a mouse model of septic cardiomyopathy.^46^ To investigate whether T‐CA may also regulate oxidative stress levels to exert a modulatory impact, this study examined oxidative stress indicators in cellular models and SAKI mice. T‐CA elevated the levels of antioxidant indexes SOD and GSH while reducing ROS and MDA, which suggests that T‐CA might alleviate SAKI by reducing the inflammation and oxidative stress levels.

During sepsis, elevated ROS levels trigger mitochondrial damage, including decreased MMP, inadequate ATP supply, changes that impair mitochondrial function and contribute to the production of more ROS.^47^ As an indispensable component of metabolic reprogramming, normal mitochondrial function is essential for maintaining the level of oxidative phosphorylation in the body.^10^, ^48^, ^49^ It has been found that mitochondria protect normal cellular function by optimizing energy expenditure, inhibiting pro‐apoptotic triggers, and reprogramming substrate utilization.^50^, ^51^ However, mitochondrial dysfunction is often associated with the SAKI process.^37^ Peng et al. found that by improving mitochondrial metabolism and biogenesis was able to attenuate inflammatory response and renal tubular cell death and alleviate renal histopathological injury in SAKI mice.^52^ The present study similarly found that MMP was reduced, ATP production was diminished, and mitochondrial morphology was disrupted in SAKI mice and cellular models, whereas T‐CA treatment ameliorated mitochondrial dysfunction and could protect the integrity of mitochondrial morphology. Therefore, we can conclude that T‐CA reduces inflammatory and oxidative stress damage, improves mitochondrial dysfunction, and has a protective effect on renal injury due to SAKI.

When oxidative stress occurs, Nrf2 translocates to the nucleus, activating antioxidant response elements and initiating cellular defense mechanisms.^53^ It has been shown that when SAKI occurs, the Nrf2 pathway is inhibited, causing increased oxidative stress, and that some anti‐inflammatory substances can ameliorate SAKI by activating Nrf2 signaling.^28^ In addition, the NF‐κB signaling pathway is also crucial in SAKI. Li et al. reported that the activation of NF‐κB was observed in the kidneys of SAKI mice, and inhibition of this pathway attenuated renal injury in SAKI mice.^37^ Ni et al. reported that esculin, a coumarin derivative, attenuated LPS‐induced acute lung injury, declined inflammatory factors levels, and hindered the activation of NF‐κB pathway.^54^ Therefore, we hypothesized that T‐CA may also modulate the NF‐κB/Nrf2 pathway. In this research study, the Nrf2 pathway was inhibited and NF‐κB was activated in SAKI mice and cell models, but T‐CA activated the Nrf2 pathway and inhibited NF‐κB protein expression. Notably, both NF‐κB activators and Nrf2 inhibitors attenuated the protective effects of T‐CA in SAKI mice and cellular models, suggesting that T‐CA acts through modulating the NF‐κB/Nrf2 pathway. Studies have found that the main way metabolites act is by directly binding to proteins and modulating biological functions.^55^ As reported in Nature, 3‐oxoLCA suppresses Th17 cell differentiation through interacting with the RORγt ligand binding domain.^56^ Meanwhile, we also noted that both subunits of T‐CA: coumarin and acetic acid have similar biological activities,^57^ suggesting that T‐CA may also be involved in SAKI progression through binding to target proteins. We found that T‐CA down‐regulated the expression of GRK5 in SAKI mice and cellular models, whereas overexpression of GRK5 reduced the protective impact of T‐CA in the SAKI model, confirming that T‐CA acts by regulating GRK5. Notably, overexpression of GRK5 promoted NF‐κB activation and inhibited the Nrf2 pathway, suggesting that GRK5 modulates the NF‐κB/Nrf2 pathway. To sum up, our study confirms that T‐CA ameliorates SAKI injury through modulating GRK5/NF‐κB/Nrf2 pathway.

## 5 CONCLUSION

In summary, T‐CA down‐regulated GRK5, inhibited NF‐κB activation and activated the Nrf2 pathway, which in turn ameliorated the LPS‐induced HK‐2 cell injury, attenuated renal injury and mitochondrial dysfunction in SAKI mice. This study elucidated that T‐CA has therapeutic potential for SAKI and demonstrated that down‐regulation of GRK5 can effectively ameliorate SAKI injury, providing a promising target for the SAKI therapy. However, there are some shortcomings in this study. Due to the limited experimental conditions, the therapeutic effect of T‐CA was not evaluated in the long term in this study, and further in vivo exploration of the long‐term therapeutic impact of T‐CA on SAKI in animals and evaluation of the safety of T‐CA are needed in future investigations. In addition, this research study suffers from a limited sample size, and future investigations need to rise the sample size.

## AUTHOR CONTRIBUTIONS

Jie Liu developed and planned the study, performed experiments, and interpreted results. Edited and refined the manuscript with a focus on critical intellectual contributions. Yugang Deng and Kunyang Lei participated in collecting, assessing, and interpreting the data. Made significant contributions to data interpretation and manuscript preparation. Yaqi Li and Siwei Ma provided substantial intellectual input during the drafting and revision of the manuscript. The final version of the manuscript has been reviewed and approved by all authors.

## CONFLICT OF INTEREST STATEMENT

The authors declare no conflicts of interest.

## ETHICS STATEMENT

This research was approved by Henan Provincial People's Hospital Ethics Committee.

## CONSENT TO PUBLISH

The manuscript has neither been previously published nor is under consideration by any other journal. The authors have all approved the content of the paper.

## Data Availability

The data supporting the findings of this study can be obtained from the corresponding author, Jie Liu, upon request.

## References

[1] Liu, D. , S. Y. Huang , J. H. Sun , H. C. Zhang , Q. L. Cai , C. Gao , Li Li , et al. 2022. “Sepsis‐Induced Immunosuppression: Mechanisms, Diagnosis and Current Treatment Options.” Military Medical Research 9(1): 56. 10.1186/s40779-022-00422-y. 36209190 PMC9547753

[2] Reinhart, K. , R. Daniels , N. Kissoon , F. R. Machado , R. D. Schachter , and S. Finfer . 2017. “Recognizing Sepsis as a Global Health Priority – A WHO Resolution.” New England Journal of Medicine 377(5): 414–417. 10.1056/NEJMp1707170. 28658587

[3] Caraballo, C. , and F. Jaimes . 2019. “Organ Dysfunction in Sepsis: An Ominous Trajectory From Infection to Death.” Yale Journal of Biology and Medicine 92(4): 629–640. 31866778 PMC6913810

[4] Pais, T. , S. Jorge , and J. A. Lopes . 2024. “Acute Kidney Injury in Sepsis.” International Journal of Molecular Sciences 25(11): 5924. 10.3390/ijms25115924. 38892111 PMC11172431

[5] Vincent, J. L. 2022. “Current Sepsis Therapeutics.” EBioMedicine 86: 104318. 10.1016/j.ebiom.2022.104318. 36470828 PMC9782815

[6] Hellman, T. , P. Uusalo , and M. J. Järvisalo . 2021. “Renal Replacement Techniques in Septic Shock.” International Journal of Molecular Sciences 22(19): 10238. 10.3390/ijms221910238. 34638575 PMC8508758

[7] Ahmadian, E. , S. M. Hosseiniyan Khatibi , S. Razi Soofiyani , S. Abediazar , M. M. Shoja , M. Ardalan , and Sepideh Zununi Vahed . 2021. “Covid‐19 and Kidney Injury: Pathophysiology and Molecular Mechanisms.” Reviews in Medical Virology 31(3): e2176. 10.1002/rmv.2176. 33022818 PMC7646060

[8] Balkrishna, A. , S. Sinha , A. Kumar , V. Arya , A. K. Gautam , M. Valis , Kamil Kuca , D. Kumar , and R. Amarowicz . 2023. “Sepsis‐Mediated Renal Dysfunction: Pathophysiology, Biomarkers and Role of Phytoconstituents in Its Management.” Biomedicine & Pharmacotherapy 165: 115183. 10.1016/j.biopha.2023.115183. 37487442

[9] Wu, H. , H. Huang , and Y. Zhao . 2023. “Interplay Between Metabolic Reprogramming and Post‐Translational Modifications: From Glycolysis to Lactylation.” Frontiers in Immunology 14: 1211221. 10.3389/fimmu.2023.1211221. 37457701 PMC10338923

[10] Liu, C. , W. Wei , Y. Huang , P. Fu , L. Zhang , and Y. Zhao . 2024. “Metabolic Reprogramming in Septic Acute Kidney Injury: Pathogenesis and Therapeutic Implications.” Metabolism 158: 155974. 10.1016/j.metabol.2024.155974. 38996912

[11] Kounatidis, D. , N. G. Vallianou , S. Psallida , F. Panagopoulos , E. Margellou , D. Tsilingiris , I. Karampela , T. Stratigou , and M. Dalamaga . 2024. “Sepsis‐Associated Acute Kidney Injury: Where Are We Now?” Medicina (Kaunas) 60(3): 434. 10.3390/medicina60030434. 38541160 PMC10971830

[12] Ji, R. , W. Chen , Y. Wang , F. Gong , S. Huang , M. Zhong , Z. Liu , et al. 2021. “The Warburg Effect Promotes Mitochondrial Injury Regulated by Uncoupling Protein‐2 in Septic Acute Kidney Injury.” Shock 55(5): 640–648. 10.1097/shk.0000000000001576. 32496419

[13] Manrique‐Caballero, C. L. , G. Del Rio‐Pertuz , and H. Gomez . 2021. “Sepsis‐Associated Acute Kidney Injury.” Critical Care Clinics 37(2): 279–301. 10.1016/j.ccc.2020.11.010. 33752856 PMC7995616

[14] Chatzakos, V. , K. Slätis , T. Djureinovic , T. Helleday , and M. C. Hunt . 2012. “N‐Acyl Taurines Are Anti‐Proliferative in Prostate Cancer Cells.” Lipids 47(4): 355–361. 10.1007/s11745-011-3639-9. 22160494

[15] Waluk, D. P. , K. Vielfort , S. Derakhshan , H. Aro , and M. C. Hunt . 2013. “N‐Acyl Taurines Trigger Insulin Secretion by Increasing Calcium Flux in Pancreatic β‐Cells.” Biochemical and Biophysical Research Communications 430(1): 54–59. 10.1016/j.bbrc.2012.11.026. 23159632

[16] Grevengoed, T. J. , S. A. J. Trammell , M. K. McKinney , N. Petersen , R. L. Cardone , J. S. Svenningsen , D. Ogasawara , et al. 2019. “N‐Acyl Taurines Are Endogenous Lipid Messengers That Improve Glucose Homeostasis.” Proceedings of the National Academy of Sciences of the United States of America 116(49): 24770–24778. 10.1073/pnas.1916288116. 31740614 PMC6900532

[17] Guo, Y. , H. Zhang , Z. Lv , Y. Du , D. Li , H. Fang , J. You , L. Yu , and R. Li . 2023. “Up‐Regulated CD38 by Daphnetin Alleviates Lipopolysaccharide‐Induced Lung Injury via Inhibiting MAPK/NF‐κB/NLRP3 Pathway.” Cell Communication and Signaling: CCS 21(1): 66. 10.1186/s12964-023-01041-3. 36998049 PMC10061746

[18] Kayki‐Mutlu, G. , and W. J. Koch . 2023. “Novel Roles for G Protein‐Coupled Receptor Kinases in Cardiac Injury and Repair.” Biochemical Society Transactions 51(2): 715–724. 10.1042/bst20221317. 37013982 PMC12167929

[19] de Lucia, C. , L. A. Grisanti , G. Borghetti , M. Piedepalumbo , J. Ibetti , A. M. Lucchese , E. W. Barr , et al. 2022. “G Protein‐Coupled Receptor Kinase 5 (GRK5) Contributes to Impaired Cardiac Function and Immune Cell Recruitment in Post‐Ischemic Heart Failure.” Cardiovascular Research 118(1): 169–183. 10.1093/cvr/cvab044. 33560342 PMC8752360

[20] Xiang, H. , J. Huang , A. Song , F. Liu , J. Xiong , and C. Zhang . 2024. “GRK5 Promoted Renal Fibrosis via HDAC5/Smad3 Signaling Pathway.” The FASEB Journal 38(2): e23422. 10.1096/fj.202301595RRR. 38206179

[21] Toya, M. , Y. Akasaki , T. Sueishi , I. Kurakazu , M. Kuwahara , T. Uchida , T. Tsutsui , et al. 2021. “G Protein‐Coupled Receptor Kinase 5 Deletion Suppresses Synovial Inflammation in a Murine Model of Collagen Antibody‐Induced Arthritis.” Scientific Reports 11(1): 10481. 10.1038/s41598-021-90020-0. 34006987 PMC8131379

[22] Xu, M. , Y. Shao , K. Lin , Y. Liu , Y. Lin , Y. Lin , R. Yang , et al. 2023. “Genetic Arg‐304‐His Substitution in GRK5 Protects Against Sepsis Progression by Alleviating NF‐κB‐Mediated Inflammation.” International Immunopharmacology 115: 109629. 10.1016/j.intimp.2022.109629. 36584571

[23] Yu, H. , L. Lin , Z. Zhang , H. Zhang , and H. Hu . 2020. “Targeting NF‐κB Pathway for the Therapy of Diseases: Mechanism and Clinical Study.” Signal Transduction and Targeted Therapy 5(1): 209. 10.1038/s41392-020-00312-6. 32958760 PMC7506548

[24] Choi, M. C. , J. Jo , J. Park , H. K. Kang , and Y. Park . 2019. “NF‐κB Signaling Pathways in Osteoarthritic Cartilage Destruction.” Cells 8(7): 734. 10.3390/cells8070734. 31319599 PMC6678954

[25] He, F. , X. Ru , and T. Wen . 2020. “NRF2, a Transcription Factor for Stress Response and Beyond.” International Journal of Molecular Sciences 21(13): 4777. 10.3390/ijms21134777. 32640524 PMC7369905

[26] Jin, W. , Y. Xue , Y. Xue , X. Han , Q. Song , J. Zhang , Z. Li , et al. 2020. “Tannic Acid Ameliorates Arsenic Trioxide‐Induced Nephrotoxicity, Contribution of NF‐κB and Nrf2 Pathways.” Biomedicine & Pharmacotherapy 126: 110047. 10.1016/j.biopha.2020.110047. 32146384

[27] Sueishi, T. , Y. Akasaki , N. Goto , I. Kurakazu , M. Toya , M. Kuwahara , T. Uchida , et al. 2020. “GRK5 Inhibition Attenuates Cartilage Degradation via Decreased NF‐κB Signaling.” Arthritis & Rheumatology 72(4): 620–631. 10.1002/art.41152. 31696655

[28] Xu, L. , J. Cai , C. Li , M. Yang , T. Duan , Q. Zhao , Y. Xi , et al. 2023. “4‐Octyl Itaconate Attenuates LPS‐Induced Acute Kidney Injury by Activating Nrf2 and Inhibiting STAT3 Signaling.” Molecular Medicine 29(1): 58. 10.1186/s10020-023-00631-8. 37095432 PMC10127401

[29] Wu, H. , R. Wu , T. Liu , H. Ma , G. Xue , and M. Liu . 2023. “Peroxiredoxin 6 Alleviates High Glucose‐Induced Inflammation and Apoptosis in HK‐2 Cells by Inhibiting TLR4/NF‐κB Signaling.” Annals of Translational Medicine 11(2): 41. 10.21037/atm-22-6063. 36819569 PMC9929773

[30] Dong, J. , M. Liu , Y. Bian , W. Zhang , C. Yuan , D. Wang , Z. Zhou , Y. Li , and Y. Shi . 2024. “MicroRNA‐204‐5p Ameliorates Renal Injury via Regulating Keap1/Nrf2 Pathway in Diabetic Kidney Disease.” Diabetes, Metabolic Syndrome and Obesity 17: 75–92. 10.2147/dmso.S441082. PMC10775805 38196512

[31] Zhang, S. , J. Ma , L. Sheng , D. Zhang , X. Chen , J. Yang , and D. Wang . 2017. “Total Coumarins From Hydrangea paniculata Show Renal Protective Effects in Lipopolysaccharide‐Induced Acute Kidney Injury via Anti‐inflammatory and Antioxidant Activities.” Frontiers in Pharmacology 8: 872. 10.3389/fphar.2017.00872. 29311915 PMC5735979

[32] Jin, Y. , J. Qian , X. Ju , X. Bao , L. Li , S. Zheng , X. Chen , et al. 2018. “Osthole Protects against Acute Lung Injury by Suppressing NF‐κB‐Dependent Inflammation.” Mediators of Inflammation 2018: 4934592. 10.1155/2018/4934592. 30057486 PMC6051001

[33] Gao, M. , H. Li , Q. Liu , N. Ma , P. Zi , H. Shi , and Y. Du . 2022. “KLF6 Promotes Pyroptosis of Renal Tubular Epithelial Cells in Septic Acute Kidney Injury.” Shock 57(3): 417–426. 10.1097/shk.0000000000001881. 34710881

[34] Lin, Z. , J. Jin , and X. Shan . 2019. “Fish Oils Protects against Cecal Ligation and Puncture‐Induced Septic Acute Kidney Injury via the Regulation of Inflammation, Oxidative Stress and Apoptosis.” International Journal of Molecular Medicine 44(5): 1771–1780. 10.3892/ijmm.2019.4337. 31545434 PMC6777667

[35] Cai, F. , D. Li , Y. Xie , X. Wang , H. Ma , H. Xu , J. Cheng , H. Zhuang , and Z.‐C. Hua . 2024. “Sulfide:Quinone Oxidoreductase Alleviates Ferroptosis in Acute Kidney Injury via Ameliorating Mitochondrial Dysfunction of Renal Tubular Epithelial Cells.” Redox Biology 69: 102973. 10.1016/j.redox.2023.102973. 38052107 PMC10746537

[36] Wang, P. , J. Huang , Y. Li , R. Chang , H. Wu , J. Lin , and Z. Huang . 2015. “Exogenous Carbon Monoxide Decreases Sepsis‐Induced Acute Kidney Injury and Inhibits NLRP3 Inflammasome Activation in Rats.” International Journal of Molecular Sciences 16(9): 20595–20608. 10.3390/ijms160920595. 26334271 PMC4613220

[37] Li, J. , L. Wang , B. Wang , Z. Zhang , L. Jiang , Z. Qin , Y. Zhao , and B. Su . 2023. “NOX4 Is a Potential Therapeutic Target in Septic Acute Kidney Injury by Inhibiting Mitochondrial Dysfunction and Inflammation.” Theranostics 13(9): 2863–2878. 10.7150/thno.81240. 37284448 PMC10240817

[38] Tang, Y. , H. Luo , Q. Xiao , L. Li , X. Zhong , J. Zhang , F. Wang , G. Li , L. Wang , and Y. Li . 2021. “Isoliquiritigenin Attenuates Septic Acute Kidney Injury by Regulating Ferritinophagy‐Mediated Ferroptosis.” Renal Failure 43(1): 1551–1560. 10.1080/0886022x.2021.2003208. 34791966 PMC8604484

[39] Wang, Y. , W. Xi , X. Zhang , X. Bi , B. Liu , X. Zheng , and X. Chi . 2022. “CTSB Promotes Sepsis‐Induced Acute Kidney Injury Through Activating Mitochondrial Apoptosis Pathway.” Frontiers in Immunology 13: 1053754. 10.3389/fimmu.2022.1053754. 36713420 PMC9880165

[40] Chang, Y. M. , Y. T. Chou , W. C. Kan , and C. C. Shiao . 2022. “Sepsis and Acute Kidney Injury: A Review Focusing on the Bidirectional Interplay.” International Journal of Molecular Sciences 23(16): 9159. 10.3390/ijms23169159. 36012420 PMC9408949

[41] Mao, Y. , F. Jiang , X. J. Xu , L. B. Zhou , R. Jin , L. L. Zhuang , C.‐X. Juan , and G.‐P. Zhou . 2023. “Inhibition of IGF2BP1 Attenuates Renal Injury and Inflammation by Alleviating m6A Modifications and E2F1/MIF Pathway.” International Journal of Biological Sciences 19(2): 593–609. 10.7150/ijbs.78348. 36632449 PMC9830505

[42] Ronco, C. , R. Bellomo , and J. A. Kellum . 2019. “Acute Kidney Injury.” Lancet 394(10212): 1949–1964. 10.1016/s0140-6736(19)32563-2. 31777389

[43] Xu, Z. , X. Wang , W. Kuang , S. Wang , and Y. Zhao . 2023. “Kaempferol Improves Acute Kidney Injury via Inhibition of Macrophage Infiltration in Septic Mice.” Bioscience Reports 43(7). 10.1042/bsr20230873. PMC10372469 37440431

[44] Ow, C. P. C. , A. Trask‐Marino , A. H. Betrie , R. G. Evans , C. N. May , and Y. R. Lankadeva . 2021. “Targeting Oxidative Stress in Septic Acute Kidney Injury: From Theory to Practice.” Journal of Clinical Medicine 10(17): 3798. 10.3390/jcm10173798. 34501245 PMC8432047

[45] Zhao, Q. , R. Zhang , Y. Wang , T. Li , J. Xue , and Z. Chen . 2024. “FOXQ1, Deubiquitinated by USP10, Alleviates Sepsis‐Induced Acute Kidney Injury by Targeting the CREB5/NF‐κB Signaling axis.” Biochimica et Biophysica Acta, Molecular Basis of Disease 1870(7): 167331. 10.1016/j.bbadis.2024.167331. 38960057

[46] Su, Z. , M. Gao , L. Weng , and T. Xu . 2024. “Esculin Targets TLR4 to Protect Against LPS‐Induced Septic Cardiomyopathy.” International Immunopharmacology 131: 111897. 10.1016/j.intimp.2024.111897. 38513575

[47] Wu, M. , Z. Huang , P. D. P. Akuetteh , Y. Huang , and J. Pan . 2024. “Eriocitrin Prevents Sepsis‐Induced Acute Kidney Injury Through Anti‐Inflammation and Anti‐Oxidation via Modulating Nrf2/DRP1/OPA1 Signaling Pathway.” Biochimica et Biophysica Acta (BBA) ‐ General Subjects 1868(7): 130628. 10.1016/j.bbagen.2024.130628. 38642815

[48] Luther, T. , S. Bülow‐Anderberg , P. Persson , S. Franzén , P. Skorup , A. Wernerson , K. Hultenby , F. Palm , T. A. Schiffer , and R. Frithiof . 2023. “Renal Mitochondrial Dysfunction in Ovine Experimental Sepsis‐Associated Acute Kidney Injury.” American Journal of Physiology ‐ Renal Physiology 324(6): F571–F580. 10.1152/ajprenal.00294.2022. 37102685

[49] Acuña‐Castroviejo, D. , I. Rahim , C. Acuña‐Fernández , M. Fernández‐Ortiz , J. Solera‐Marín , R. K. A. Sayed , M. E. Díaz‐Casado , I. Rusanova , L. C. López , and G. Escames . 2017. “Melatonin, Clock Genes and Mitochondria in Sepsis.” Cellular and Molecular Life Sciences 74(21): 3965–3987. 10.1007/s00018-017-2610-1. 28785808 PMC11107653

[50] Gómez, H. , J. A. Kellum , and C. Ronco . 2017. “Metabolic Reprogramming and Tolerance During Sepsis‐Induced AKI.” Nature Reviews Nephrology 13(3): 143–151. 10.1038/nrneph.2016.186. 28090081 PMC5508527

[51] Sun, J. , J. Zhang , J. Tian , G. M. Virzì , K. Digvijay , L. Cueto , Y. Yin , M. H. Rosner , and C. Ronco . 2019. “Mitochondria in Sepsis‐Induced AKI.” Journal of the American Society of Nephrology 30(7): 1151–1161. 10.1681/asn.2018111126. 31076465 PMC6622414

[52] Peng, X. , S. Chen , Y. Wang , M. Jin , F. Mei , Y. Bao , X. Liao , Y. Chen , and W. Gong . 2022. “SGLT2i Reduces Renal Injury by Improving Mitochondrial Metabolism and Biogenesis.” Molecular Metabolism: 101613. 10.1016/j.molmet.2022.101613. 36241142

[53] Ulasov, A. V. , A. A. Rosenkranz , G. P. Georgiev , and A. S. Sobolev . 2022. “Nrf2/Keap1/ARE Signaling: Towards Specific Regulation.” Life Sciences 291: 120111. 10.1016/j.lfs.2021.120111. 34732330 PMC8557391

[54] Ni, J. , G. Li , N. Dai , Z. Quan , H. Tong , and Y. Liu . 2023. “Esculin Alleviates LPS‐Induced Acute Lung Injury via Inhibiting Neutrophil Recruitment and Migration.” International Immunopharmacology 119: 110177. 10.1016/j.intimp.2023.110177. 37068336 PMC10105132

[55] Piazza, I. , K. Kochanowski , V. Cappelletti , T. Fuhrer , E. Noor , U. Sauer , and P. Picotti . 2018. “A Map of Protein‐Metabolite Interactions Reveals Principles of Chemical Communication.” Cell 172(1–2): 358–372.e23. 10.1016/j.cell.2017.12.006. 29307493

[56] Hang, S. , D. Paik , L. Yao , E. Kim , J. Trinath , J. Lu , S. Ha , et al. 2019. “Bile Acid Metabolites Control T(H)17 and T(reg) Cell Differentiation.” Nature 576(7785): 143–148. 10.1038/s41586-019-1785-z. 31776512 PMC6949019

[57] Chan, C. Y. , I. Singh , H. Magnuson , M. Zohaib , K. P. Bakshi , B. Le François , A. Anazco‐Ayala , et al. 2015. “Taurine Targets the GluN2b‐Containing NMDA Receptor Subtype.” Advances in Experimental Medicine & Biology 803: 531–544. 10.1007/978-3-319-15126-7_43. 25833525

